# An exploration of key issues and potential solutions that impact physician wellbeing and professional fulfillment at an academic center

**DOI:** 10.7717/peerj.1783

**Published:** 2016-03-10

**Authors:** Iris Schrijver, Keri J.S. Brady, Mickey Trockel

**Affiliations:** 1 Department of Pathology, Stanford University School of Medicine, Stanford, CA, United States; 2 Department of Pediatrics, Stanford University School of Medicine, Stanford, CA, United States; 3 Department of Health Law, Policy & Management, Boston University School of Public Health, Boston, MA, United States; 4 Department of Psychiatry & Behavioral Sciences, Stanford University School of Medicine, Stanford, CA, United States

**Keywords:** Physician wellness, Professional fulfillment, Dissatisfaction, Burnout, Work-life integration

## Abstract

**Background.** Physician wellness is a vital element of a well-functioning health care system. Not only is physician wellness empirically associated with quality and patient outcomes, but its ramifications span individual, interpersonal, organizational, and societal levels. The purpose of this study was to explore academic physicians’ perceptions about their work-related wellness, including the following questions: (a) What are the workplace barriers and facilitators to their wellness? (b) What workplace solutions do theythinkwouldimprove their wellness? (c)What motivates their work? and (d) What existing wellness programs are they aware of?

**Methods.** A multi-method design was applied to conduct a total of 19 focus group sessions in 17 clinical departments. All academic faculty ranks and career lines were represented in the 64 participating physicians, who began the sessions with five open-ended survey questions pertaining to physician wellness in their work environment. Participants entered their answers into a web-based survey program that enabled anonymous data collection. The initial survey component was followed by semi-structured focus group discussion. Data analysis of this qualitative study was informed by the general inductive approach as well as a review of extant literature through September 2015 on physician wellness, professional fulfillment, satisfaction, dissatisfaction, burnout and work-life.

**Results.** Factors intrinsic to the work of physicians dominated the expressed reasons for work motivation. These factors all related to the theme of overall contribution, with categories of meaningful work, patient care, teaching, scientific discovery, self-motivation and matching of career interests. Extrinsic factors such as perceptions of suboptimal goal alignment, inadequate support, restricted autonomy, lack of appreciation, and suboptimal compensation and benefits dominated the risk of professional dissatisfaction.

**Discussion.** Our findings indicate that the factors that enhance professional fulfillment and those that precipitate burnout are distinct: motivation and quality of work performed were supported by domains intrinsic to the work itself, whereas external dysfunctional work aspects resulted in frustration. Thus, it can be anticipated that optimization of physician wellness would require tailored approaches in each of these dimensions with sustained funding and support for wellness initiatives. Physicians identified the availability of resources to enable them to thrive and provide excellent patient care as their most important wellness-enhancing factor.

## Introduction

Wellness includes not just the absence of distress or illness but also emotional wellbeing, physical health, and social relationships. It allows physicians to prosper in the personal and professional dimensions of their lives (Shanafelt, Sloan & Habermann, 2003; Wallace, Lemaire & Ghali, 2009). Professional fulfillment, a construct we conceptualize within the positive domain of physician wellness, includes dimensions of happiness, self-worth, self-efficacy, and satisfaction at work. Physicians can practice in multiple settings and in a variety of professional roles, but their service to patients and alleviation of suffering are typically perceived to be the central and most fulfilling aspects of their work. Other activities physicians consider rewarding include personal interactions with patients and colleagues, teaching, and scholarship (Shanafelt et al., 2009). It follows that professional fulfillment can be achieved in multiple ways, depending on the individual physician. It may be enhanced by creative growth, continued learning, and productive contribution (Shanafelt, Sloan & Habermann, 2003; Wallace, Lemaire & Ghali, 2009).

In recent decades, physician wellness has diminished in almost every aspect of professional life (Murray et al., 2001; Spickard, Gabbe & Christensen, 2002), while concomitantly burnout symptoms, which represent indicators of the absence of work-related wellness, have increased. The percentage of physicians with burnout symptoms ranges from 30 to 68% across medical specialties (Williams et al., 2010), and burnout is most prevalent in urgent care specialties (Shanafelt et al., 2012). Outside the US, burnout affects between 25 and 60% of practicing physicians (Wallace, Lemaire & Ghali, 2009), but percentages are population dependent and studies vary by size, metrics, methods, and emphasis. Resulting from long-term stress at work, burnout is a syndrome characterized by three distinct domains, and is commonly evaluated with the Maslach Burnout Inventory (MBI) (Maslach, Jackson & Leiter, 1996; Maslach, Schaufeli & Leiter, 2001; Schaufeli et al., 2001; Shanafelt et al., 2012). The three domains include: (1) “Emotional exhaustion,” reflecting feeling drained or loss of enthusiasm for work; (2) “Depersonalization,” which can manifest as cynicism or uncaring behavior towards others; and (3) a “Low sense of personal accomplishment,” associated with a perceived loss of meaning in the work and feelings of ineffectiveness (Maslach, Jackson & Leiter, 1996; Shanafelt et al., 2002; Spickard, Gabbe & Christensen, 2002). The latter domain appears to suboptimally reflect burnout symptoms in physicians. In this population, therefore, greater emphasis is awarded to scores on the “Emotional exhaustion” and “Depersonalization” scale (Maslach, Jackson & Leiter, 1996; Shanafelt et al., 2012; Dyrbye et al., 2013).

The inaugural physician wellness survey from the Stanford Committee for Professional Satisfaction and Support (SCPSS) (http://wellmd.stanford.edu/content/dam/sm/wellmd/documents/med-staff-survey-report-4-2014.pdf) identified that drivers of professional fulfillment at this academic center included perceived appreciation, peer support, alignment of institutional goals with personal values, and schedule control. Most physicians (76% of 813 participants) reported at least moderate professional fulfillment, based on an assessment of happiness, self-worth, self-efficacy, and satisfaction at work. It also illuminated that 26% of participating physicians had symptoms of burnout, based on their own assessment of whether their degree of symptoms (such as emotional exhaustion and depersonalization) would qualify as burnout. These were particularly prevalent among physicians in the Clinician Educator faculty line, focused on clinical service and teaching, and among Medical Center Line faculty, who have a broad mix of duties including clinical service, teaching, scholarship, and administration. The survey indicated that sleep-related impairment, lack of schedule control and lack of perceived mission alignment were important risk factors for burnout.

The study reported here built on the initial physician-wellness survey and aimed to explore university-employed physicians’ perceptions of work-related wellness. Burnout, which can be viewed as an indicator of the absence of work-related wellness, is an important consideration in any such exploration. Specific aims of our investigation were to explore physicians’ (a) perceived workplace barriers and facilitators to physician wellness; (b) proposed solutions to work-related wellness barriers; (c) sources of work motivation; and (d) awareness of existing organizational wellness programs. The study’s goal was to achieve a refined understanding of physician work-life wellness optimization needs, in order to reduce the risk of burnout. It was conducted to illuminate concerns that could be addressed in the short or medium-term and, if improved, would be expected to make a substantial and positive difference. In addition, the findings of this study were intended to inform the next Stanford physician wellness survey, in which all members of the medical staff would be invited to participate. With the support of SCPSS and Stanford Medicine leadership, that survey’s findings are anticipated to guide the formulation of actions that can effectively improve alignment of physician health promotion opportunities and organizational goals. The more general findings from this study and the survey are envisioned to inform and support the long-term Stanford Medicine commitment to improving professional fulfillment among physicians. They should also be considered in the context of existing literature, in which positive and negative circumstances associated with physician wellbeing, their effects, and the importance of cultivating resilience have been described (Linzer et al., 2000; Arnetz, 2001; Spickard, Gabbe & Christensen, 2002; Shanafelt, Sloan & Habermann, 2003; Wallace & Lemaire, 2007; Halbesleben & Rathert, 2008; Linzer et al., 2009; Schloss et al., 2009; Wallace, Lemaire & Ghali, 2009; Williams et al., 2010; Eley et al., 2013; Salles, Liebert & Greco, 2015). The current study supports findings from earlier literature and reveals a path towards comprehensive, multipronged optimization of physician wellness, as suggested by physicians in their own words.

## Materials and Methods

### Study population

This study was conducted at Stanford University. It included 64 participating physicians from the 17 clinical departments of Stanford Medicine, which includes Stanford Health Care and Stanford Children’s Health, serving the adult and pediatric patient populations, respectively. Physicians from 17 departments (Anesthesia, Dermatology, Medicine, Neurology and Neurological sciences, Neurosurgery, Obstetrics and Gynecology, Ophthalmology, Orthopedic surgery, Otolaryngology, Pathology, Pediatrics, Psychiatry, Radiation Oncology, Radiology, Surgery, Cardiothoracic Surgery, Urology) were eligible for participation if they had an M.D. or D.O degree, if they were in any of the Stanford career trajectories (faculty lines), and if they were members of the medical staff involved in patient care at the adult hospital, the children’s hospital, or both, including associated clinics. Peripheral sites of the greater network of the University Healthcare Alliance (UHA) were excluded. Physicians with trainee status were also excluded.

### Design and methods

This multi-method study included two design components: a brief, electronic open-ended survey followed by a peer-to-peer focus group. A list of all Stanford physicians was utilized as a sampling frame from which initial purposive sampling, stratified by department of all 17 clinical departments, occurred. Additional participants were recruited based on recommendations by physician colleagues approached by the primary investigator (I.S.), a peer physician practicing at Stanford Medicine. Referral to alternative or additional physicians was allowed. Invitations to participate in a brief in-person, online survey and a “peer-to-peer focus group” were sent via email to all sampled physicians. The focus groups were envisioned to be relatively small in order to be able to achieve greater depth of discussion. On average, eight physicians were approached per department, with a response rate of ∼46% (64/140). Each department was provided with a specific date and time to participate in the study. If they were unable to participate in their department’s designated focus group session, but expressed interest in a separate meeting time, that opportunity was provided. Only three physicians participated by this mechanism and formed a separate focus group, which may indicate that general time constraints (prioritization of tasks at hand) as well as an unwillingness to participate in a wellness discussion were the main reasons to decline. The remaining 61 participants engaged in focus groups arranged per clinical department.

Because one department was split into two focus groups for concept piloting and location reasons, 19 focus groups were ultimately conducted, representing participants from 17 departments. The potential for participation bias is present in any relatively small study that includes optional participation. This possibility was minimized, however, by basing recruitment on suggestions by various physicians from the 17 clinical departments. It must be acknowledged that physicians who chose to participate versus those who did not may have been motivated by higher levels of wellness or by lower ones. The perceptions of personal well-being, however, although not quantified, appeared to be distributed fairly evenly among participants, reflecting a continuum rather than one end of the spectrum. All ranks and academic faculty lines were invited and ultimately represented, with a gender balance of 28 male and 36 female physicians. Specific faculty ranks (assistant professor, associate professor, or full professor) or faculty lines were not recorded for confidentiality reasons in the relatively small individual focus groups. However, there was an essentially equal distribution of faculty ranks, with a preponderance of participants in those faculty lines with the most clinical service. The Stanford faculty line that emphasizes basic science and usually includes an only small clinical component was under-represented as we had only one such participant. Attendance per department had a mean of 3.4 and a median of three individuals. The focus groups were held between March and May of 2015. Notes were reviewed and integrated within hours to a few days after each focus group session.

**Figure 1:**
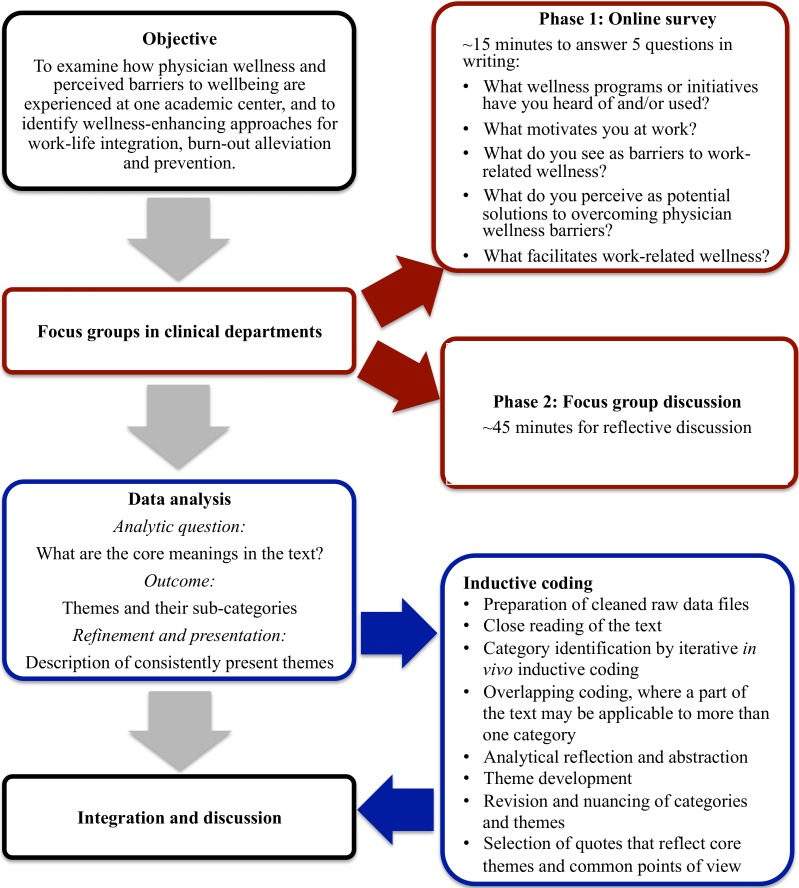
Schematic overview of the multi-method study with general inductive data analysis. The vertical pathway illustrates the data collection and analysis steps. The supplemental information on the right illustrates process details and the iterative nature of the general inductive data analysis (based on Thomas, 2006) with final integration of the data and report generation.

Bias could arise in a discussion format. In this study, however, that risk was reduced by the study design. To encourage independent and unbiased focus group discussions, prior to the start of each forty-five minute focus group discussion, participants were asked to take 15 min to independently complete an open-ended, five-question online survey. Focus group questions in the online survey were informed by results on wellness and burnout from the initial Stanford Physician Wellness Survey, which was administered in 2013, and by the need to evaluate effective reach of currently available physician wellness programs. Anonymous survey responses were collected via Qualtrics (http://www.qualtrics.com). For participants who did not bring an electronic device or chose to not use their own, devices were made available at the meetings. The survey questions were: (1) What wellness programs or initiatives at Stanford have you heard of and/or used?; (2) What motivates you at work?; (3) What do you see as barriers to work-related wellness at Stanford?; (4) What do you perceive as potential solutions to overcoming physician wellness barriers at Stanford?; (5) What facilitates work-related wellness at Stanford? This initial survey component was followed by semi-structured, open-ended discussion about the issues that emerged when participants considered physician wellness at Stanford (Fig. 1). After completion of the focus group, participants without exception consented to having their direct quotes used by the facilitator, in an unattributed manner.

### Collection and analysis of qualitative data

In order to stimulate an atmosphere of confidentiality, focus group conversations were not recorded. This decision was based on initial discussion with physicians of various departments, who expressed reservations and indicated that recording would likely compromise participation and result in guarded answers. Instead, qualitative notes were taken by the focus group facilitator (I.S.) and, in 9/19 focus groups sessions (47%), by an additional research assistant (K. J. S. B.). The jointly documented sessions were followed by immediate debriefing. The feasibility of accurate note-taking by one facilitator in 10/19 groups was determined by the following: 1) during the sessions with dual note-taking, notes had high consistency between the two note-takers, serving as an internal check of accuracy 2) themes and topics raised were surprisingly similar between departments, 3) read-back to participants was used to ensure that their comments were captured as intended. At the end of each session, the facilitator reviewed the notes with the group once more to ensure accuracy and to assess whether the participants wished to add anything else. Confidentiality was maintained by elimination of personally identifying information revealed during the course of the study. The focus group responses were ultimately grouped together so that neither departments, nor individuals could be identified separately.

A semi-structured discussion environment was developed. Upon completion of the written focus group component, which typically lasted approximately 15 min, participants were asked about what came up for them during their response to the written questions. During the following conversation, participants were encouraged to describe their experience relating to each survey question and to bring up additional topics not covered in the survey that pertained to their degree of wellness in the workplace. Overall, this approach resulted in detailed descriptions. In order to maintain integrity of the comments, statements were frequently repeated back, and the conversation was summarized at the end of each session. The quality of data collection was further enhanced by methods triangulation consisting of the electronic survey, followed by the semi-structured discussion with note-taking. Researcher role and influence were documented in the notes to recognize reflexivity. Researcher triangulation was used by having the second note-taker present. Ultimately, the notes served as an audit trail of data integrity, to support and justify the development of themes, categories, and sub-categories (Table 1). Because the results for both focus group components (i.e., the five written questions and the following discussion) centered on the same themes, the data sets could be integrated after analysis.

**Table 1:** Themes and sub-categories derived from Focus Group analyses.

Intrinsic Factors	
Themes	Categories
Contribution	Meaningful work
	Patient care
	Teaching
	Discovery
Self-motivation	
Career-fit	

Extrinsic factors		
Themes	Categories	Sub-categories
Resources	Time/workload	
	Staffing	
	Inefficiencies	∙ Billing
		∙ IT/technology
		∙ Space access and facilities
	Financial support	∙ Academic support
		∙ Clinical work support
Leadership/communication	Goal alignment	∙ SHC/SCH vs. SOM
		∙ SHC/SCH and SOM vs. faculty providers
		∙ Leadership
		∙ Career fit in the context of goals alignment
	Communication	∙ Communication
		∙ Orientation
Control over the work-environment	Control	
	Autonomy	
	Performance metrics	
Connection/community	Collegiality	∙ Mentoring
	Diversity	
Work environment	Work culture	
	Facilities	∙ Physician lounge
		∙ Parking
		∙ Commuting
		∙ Offices
		∙ Disjointedness
	Wellness	∙ Awareness of wellness offerings
		∙ Wellness access issues
		∙ Perceptions around wellness
		∙ Wellness offerings for residents
		∙ Wellness offerings in industry/other institutions
Work-life integration resources	Remuneration	
	Housing	
	Child care	
	Home life	
Appreciation	Perceptions of appreciation	

**Notes.**

IT Information technology SHC Stanford Health Care SCH Stanford Children’s Health SOM School of Medicine vs Versus

Informed broadly by the critical realist paradigm (Maxwell, 2012), we applied a general inductive approach (Thomas, 2006) to the survey and focus group data analysis. Three principles underlie this approach. First, topics of investigation arise from the evaluation objectives, which guide the analysis of the data. The findings emerge directly from the raw data and are influenced by the objectives and research questions, but they are not based on models or expectations in advance. As such, the approach is distinct from deductive analysis, in which specific hypotheses are being tested. Second, raw data analysis leads to the creation of categories and a framework of analysis with central themes. Third, the results are founded on multiple raw data interpretations by the evaluators, who assign importance to individual data themes through an iterative process. Differences between evaluators could therefore exist (Thomas, 2006). This approach reflects an analytic method that mirrors that of grounded theory (Strauss & Corbin, 1998), but is more optimally suitable for the derivation of findings in the context of focused evaluation research. First, the survey responses were reviewed and organized per question, rather than per participant. Subsequently, answers for each question were grouped into emerging categories, which were refined in an iterative process. Redundancy of the categories was reduced by eliminating overlap in the process of sorting comments by similarity, resulting in a list of relative frequencies and emphasis. Key points and illustrative comments were extracted from this list. Second, the focus group discussion notes were organized similarly in a separate document, based on the categories that emerged from the survey analysis. Upon completion, categories and their overarching themes were further streamlined and refined using a constant comparative process. A summary list thereof was created, reflecting the comprehensive content, consisting of the individual comments listed for each item (Fig. 1). The most representative or revealing comments were italicized. In addition, word clouds were created for summary and presentation purposes (http://www.wordle.net).

## Results

In order to gain insight into the perceptions of academic physicians about their work-related wellness and wellness optimization needs, we queried what existing offered wellness programs they were aware of, what was motivating their work efforts, and what were perceived barriers to their wellness in the work place. We also asked what work place solutions they believed would improve their wellness. Thus, both the online and the interactive components of the focus groups enabled emergence of a broad spectrum of comments, ranging from those expressing work-related wellness to those regarding burnout symptoms. Key findings include that physicians are insufficiently aware of wellness opportunities in the work place. Where they are aware, access is often limited because of time constraints and/or location. Work motivation is driven by factors that are intrinsic to physicians and their work itself. Barriers to wellness are dominated by resource issues, and perceived limitations regarding control over the practice environment. Physicians offered a wide range of suggestions for improvement, most of which addressed extrinsic factors that contribute to dissatisfaction at work.

In the online survey, the first written question explored what wellness programs or initiatives at the institution physicians had heard of and/or used, and this was also typically the first topic brought up once group discussions began. Although BeWell@Stanford, which serves as the overarching health and wellness resource for Stanford University, emerged as the most widely known and most utilized program, the majority of participating physicians were unaware of any wellness offerings. Physicians were poorly informed about the range of available resources, and dissemination of information appeared relatively ineffective at the time of study. Moreover, physicians expressed that they had limited practical access to wellness resources, because of the time slots at which activities were offered, because of lack of protected time for such activities, and because of distance from their work location. Representative quotes illustrate this in physicians’ own voices:

∙ “I am aware of wellness programs such as a trainer available at the gym, a nutritionist available, and incentives for wellness. I have not had time to take advantage of any programs.”
∙ “I am familiar with many of their programs but unable to take advantage of any due to high work load and extremely limited flexibility of work schedule.”
∙ “Being told by a non-physician to “go for walks on my lunch hour” just illustrates the enormous chasm between my reality and the platitudes.”

The second question was designed to explore what motivated participating physicians. Factors that are intrinsic to physicians’ work itself dominated work motivation. These factors can be summarized in the unifying theme of contribution, with its categories of meaningful work, patient care, teaching, scientific discovery, self-motivation and career fit (Table 1). Thus, Stanford physicians seemed to be very well-aligned with the institutional Mission (“to care, to educate, to discover”), which is reflected in the following comments:

∙ “What motivates me at work is the same motivation that drove me to seek the medical profession: the sense that my daily work would have a positive impact on another individual and that my actions are helpful to others; hence my satisfaction is internal.”
∙ “Meaningful work. I continue to work toward achieving significant work that is both meaningful to me personally and impactful on a broader scale.”
∙ “Knowing that I am doing the best possible work for the patients.”
∙ “Making new clinical discoveries that will enhance the care of patients.”
∙ “Intellectual stimulation and the challenge of new problems.”

When asked in the third question about the barriers they perceived to work-related wellness, issues surrounding meaning of work or contribution were notably absent. Instead, physicians indicated that factors extrinsic to their immediate professional activities dominated the risk of perceived barriers to work related wellness (Table 1). Ways and means were a priority, because, as participants expressed, physicians require adequate resources to carry out their responsibilities and to provide optimal patient care. Concerns included facilitation of documentation, including the time commitment currently required for charting in the electronic medical record and for documenting billing information. Physicians also had a sense of limited control over their practice environment (scheduling, resources, autonomy) and over performance metrics: the system of performance assessment was described as having financial ramifications for individual physicians, as well as a component of public sharing of results. It was also reported, however, to take into account circumstances such as wait times and interactions with non-physician staff that were perceived to be partially, or entirely, external to the physician-patient interaction and beyond the physicians’ control. The applied performance metrics—implemented with little or no involvement of the responding focus-group physicians in the process—emerged as the most prominent factor that influenced physician dissatisfaction regarding the work place. Moreover, physicians indicated that their input was perceived to be obtained infrequently and/or not effectively adopted. In addition, physicians expressed feeling tension between hospital and university goals, because hospital initiatives were not consistently aligned with academic ones and some participants perceived that different value systems were upheld for promotion in these two work domains. In terms of facilities, the availability of a physician lounge was much appreciated, although the current facility easily could be enhanced and was unavailable to physicians who did not practice nearby. Housing, remuneration, office space, childcare and parking were other principal themes during discussion. Differences between individual departments were also brought to light, especially regarding conference travel, part-time work, and effective mentoring, although the latter appeared to have improved considerably in recent years. The descriptions below illustrate some main themes, which are listed comprehensively in Table 1:

∙ Resources: “In terms of how I see my wellness: how comfortably can I do my job in a stress-free environment? Well, that is not possible because there is stress in our jobs, but having more staff, qualified staff, and a better work environment would help.”
∙ Control: “…Workday is not reasonable. Days are frantic. So overwhelming that there is a lot of spill-over to off-service time. It is really just wanting to do your job with dignity and having recognized that you are a human being who needs to have their basic needs met, such as natural breaks within the day.”
∙ Performance metrics: “They have all these performance metrics now. And I am saying this as a person who believes in performance metrics, they can be very helpful and a useful feedback tool, but the way they implement it here is just a constant barrage of negative feedback.”
∙ Goal alignment: “Getting a balance is difficult. One feels that only academic achievements are valued but clinical work consumes most time and effort.”
∙ Autonomy: “Physicians are smart, well intentioned, and highly motivated people. They cannot stand loss of autonomy and a lot of times they receive the information passed down and we do not participate in the decision making process. It is very top down and when that happens to the Nth degree, it is a tremendous frustration.”

In the fourth question, physicians were asked to consider how to overcome barriers to work-related wellness. The broad range of potential solutions offered by physicians (Table 2) concretely addressed the extrinsic factors that contributed to their perceptions of a suboptimal working environment (Table 1). Physicians in each focus group offered ideas for improvement, some of which could be easily implemented and with minimal cost; however, physicians also voiced that they needed additional support to achieve improved work-related wellness and to optimally manage their time, their workload, and their patients. They recognized that this would require a shift to a culture in which wellness is recognized as a valid pursuit that is prioritized, which would require proactive leadership support as well as more personal attention of physicians to their wellness needs and to the wellness resources available to them. Although Table 2 details the improvements physicians suggested, the following quotes serve as direct examples:

∙ “Leadership prioritizing this [wellness] and making it part of every part of our day. Consider adding it to our mission. First to make it a priority, leadership bringing it up in meetings, departments setting up their own wellness committees that survey staff and faculty to implement wellness activities; prioritizing time for wellness and identifying space towards wellness activities. Helping staff develop work/life balance.”
∙ “When you are junior you are just overwhelmed, and close mentoring by a good mentor can make all the difference.”
∙ “Sometimes just hearing we face similar frustrations it is very therapeutic. I enjoy my colleagues. Because these interactions lead to ‘let’s work on this problem’.”
∙ “When physicians are valued and matter, then that speaks volumes and it goes towards patient care, too.”

**Table 2:** Examples of physician-suggested changes to improve physician wellness in the workplace.

Resources	
Staffing	∙ Allocation of more clinical and administrative support personnel to enable physicians to maximize their time
	∙ Consideration of different staffing models for more end of day predictability
	∙ Expansion of staffing to match work volume
Inefficiencies	∙ Analysis of basic processes and infrastructure with subsequent efficiency improvements
	∙ Documentation assistance, for example through the use of scribes
	∙ Enhancement of user-friendliness of the electronic medical record
Financial support	∙ Support for clinical work: responsiveness to physician requests for the resources that would facilitate improved patient care
	∙ Support for academic work: uniform policy for travel funds to national meetings, to present research
Leadership/communication	
Goal alignment	∙ Unified vision from the leadership of the Hospitals, the School of Medicine, and the Departments that reflects the importance of physicians taking care of themselves in order to be better be able to fulfill their roles
	∙ Ensuring that a diverse cross-section of faculty be part of strategic planning
	∙ Increasing the number of physicians in leadership roles
	∙ Creating clear career pathways and encouraging individuals to pursue their strengths
	∙ Alignment of hospital initiatives with academic ones, so that what facilitates clinical and academic promotion is congruent
Communication	∙ Increased and effective communication with, and involvement by, faculty in discussion and decisions regarding time commitments and expectations
	∙ Effective leadership and support, for example by active and effective mentoring of junior faculty, together with a proactive approach regarding appointments and promotions
	∙ Commitment to clarity and transparency with respect to availability of resources
Control over the work-environment	
Control	∙ Providing a greater degree of control over physicians’ own schedules
	∙ Increased control over the immediate clinic operations with the ability to participate in decisions, in real time
Autonomy	∙ Increased autonomy by enabling physicians to have more of a say in how they run their practice
Performance metrics	∙ Use of appropriate professional performance metrics that directly evaluate the work of the physician
Connection/community	
Collegiality	∙ Enhancement of social support in the work place, to further the role of the medical as a valuable tool for maintaining wellness through positive interactions, feedback, and encouragement
Mentoring	∙ Increasing proactive, meaningful mentorship
	∙ Providing dedicated, protected time for mentoring
	∙ Coaching to facilitate good mentoring and support of others—especially junior colleagues
	∙ Team building
Diversity	∙ Creation of and ongoing support for an inclusive environment/culture that embraces diversity
	∙ Consciousness of gender balance among decision makers and those appointed to role model functions
Work environment	
Work culture	∙ Implementing opportunities for physicians to share their experience, to take advantage of the empowering nature of such interactions
Facilities	∙ Break rooms and facilities within easy access
	∙ Protected and adequate physician space to eat and rest
	∙ Ready availability of healthy food options, such as a free salad bar
Wellness	∙ Ensuring that interactions such as focus groups can occur during the work day and need not come at a cost of personal time
	∙ Prioritization by leadership of making physician wellness part of every part of the day
	∙ Fostering an understanding that wellness is in the best interest of an institution and ultimately will increase productivity and creativity
	∙ Creating credibility of intent through visibility, by leadership bringing up the topic of wellness in meetings, by departmental wellness committees that survey faculty and staff to implement wellness activities, and prioritizing time for wellness and identifying space towards wellness activities
	∙ Providing a central source to access wellness options online, and a way to filter the things which are pertinent to oneself
	∙ Development of concrete programs that are easily accessible to physicians in different locations.
	∙ Dedicated time for wellness
	∙ A state of the art physician wellness center that has a meditation/reflection room
	∙ Exercise spaces
	∙ Discounts at gyms
	∙ Paid time off to attend a wellness activity or retreat
	∙ Incentivize physicians to participate in wellness programs to make them understand the potential benefits at a personal level
	∙ Utilization of examples from private industry, illustrating the benefits of improving employee wellness
	∙ Reduction of the number of lunch-time meetings
Appreciation	∙ Recognition of achievements of faculty in the academic as well as in the clinical realm
	∙ Making physicians feel that they are a priority and are being taken care of by providing benefits such as free parking, occasional complimentary breakfast, acknowledging good work, having social gatherings that are designed just for colleagues to interact
	∙ Tokens of appreciation such as a pass to local gyms or a percentage of a health club membership, tickets, lunches, or vouchers for hospital meals when on call
	∙ Balancing performance improvement feedback with positive feedback regarding all the things physicians are already doing
	∙ More acknowledgement of individual efforts, strengths, and creating visibility of accomplishments in patient care, research, teaching/mentoring, and administration
	∙ Vouchers for child care when child care such as the regular nanny or daycare is not available
Work-life integration resources	
Remuneration	∙ Fair compensation and housing benefits; this affects how physicians can take care of themselves, their families, and their future
Child care	∙ Provision of practically available, on-site child care, with day care hours in line with physicians’ schedules
	∙ Optimized leave policies for young parents
	∙ Free on-site back-up childcare
	∙ Alignment of holidays for the Hospitals and the School of Medicine to minimize child care issues
	∙ Scheduling of meetings with an eight-to-five meeting template
Home life	∙ Food services, laundry, dry cleaning and other domestic conveniences on site to facilitate work-life integration
	Opportunity for all physicians to earn credits towards home life assistance such as cleaning services and meal delivery^*^

**Notes.**

* Reference: Valantine & Sandborg, 2013.

The fifth written question was: “What facilitates work-related wellness at Stanford?” Upon analysis of the survey responses, it became clear that this question, as phrased, was ambiguous and therefore not answered consistently. Some physicians interpreted the question as intended and described what currently facilitated their wellness, whereas others responded with answers that described what *could* enhance work-related wellness. This question was therefore eliminated from overall analysis of the written results. Discussion group data, however, did offer insight into wellness-facilitating work aspects. These characteristics were most closely aligned with the comments pertaining to motivating work factors. Positive contributing factors included colleagues who work well as a team, having a rewarding clinical practice, the presence of opportunities and support for developing academic interests, the quality of trainees, and the realization that teaching is rewarding by helping others learn. Physicians also highlighted appreciation of good work and contributions, an environment which fosters good health practices and healthy lifestyle choices, the availability of a physician lounge, and an environment with faculty colleagues of different backgrounds to enrich the clinical and academic experience.

## Discussion

Physicians at Stanford University, which is the studied academic institution, are likely to be reasonably representative of physicians at academic institutions in the United States. They are highly motivated by their patients, their colleagues, and by the work they have the privilege to perform. However, they are not taking advantage of wellness opportunities in the work place, in part because of lack of awareness and in part because of barriers to exploring and accessing wellness offerings. Additionally, wellness in the work place is negatively affected by resource issues, suboptimal communication, perceived limited autonomy in the practice environment, a feeling of not belonging, and lack of visible appreciation (Table 1). The focus group participants provided a range of suggestions for improvement (Table 2).

One striking finding was the lack of awareness among physicians regarding available work-related wellness resources. Many physicians were aware of the general health and wellness resource of the university (BeWell@Stanford), which is proactively marketed and associated with a financial incentive for participation in certain activities. Few were cognizant of the existence of an active physician wellness committee (SCPSS) and its numerous initiatives, despite availability of a website and distribution of a monthly newsletter. Thus, it appears that physicians—literally and figuratively—will need to be reached “where they are.” Fundamentally, the majority might only become engaged by effective removal of physical and psychological barriers to wellness activities. Participating physicians also suggested a comprehensive physician-specific orientation, both for those who were newly hired and for physicians already present, which could highlight available initiatives and how these could be accessed. Physicians recognized their own role in their absence of wellness-related awareness but generally felt that time and work pressures prevented them from seeking or reading information and from active participation.

Barriers to physician wellness have been described in the professional, organizational and personal realm (Arnetz, 2001). Regarding the latter, many physicians have personality traits that foster exacting standards of responsibility, self-reliance, and prioritization of work over their own health and wellness (Arnetz, 2001; Spickard, Gabbe & Christensen, 2002; Wallace & Lemaire, 2007; Wallace, Lemaire & Ghali, 2009). Resilience, which can be viewed as a process to adapt to adversity and stress and can be influenced by an individual’s personality traits and their environment, appears critical for professional effectiveness and burnout prevention (Eley et al., 2013). An early start may be helpful: cultivating resilience and wellness in physicians in training has been suggested to be an important component of effective burnout prevention, both during the training phase and in later career stages (Salles, Liebert & Greco, 2015). In addition to personal factors, institutional culture and expectations are likely to have a substantial influence on physician wellness. For example, our results suggest that replacement of the current system of performance metrics with an alternative that would monitor physician performance in a more professionally meaningful manner would likely improve satisfaction overall and create a path to increased mission alignment, collaboration, and confidence in administrative leaders. Moreover, physician wellness could be perceived as a professional strength and as a priority if the importance of wellness were highlighted and modeled by organizational leadership. This would require consistent effort and ongoing practical support, such as exemplified by successful career customization (Valantine & Sandborg, 2013). It could also include supported and scheduled time for wellness-enhancing activities during the workday. Participating physicians almost uniformly expressed interest in improving their health and wellness but were frequently unable to envision improvement if it required an added activity to an already maximized daily schedule.

The study underscored that for physicians, work motivation, fulfillment and quality are supported by factors intrinsic to their work, whereas factors extrinsic to the nature of the work do not appear to influence well-being directly. When these are deficient, demotivation may ensue because individuals feel frustrated in their efforts or efficiency, leading to dissatisfaction (Herzberg, 2003; Brown & Gunderman, 2006). For physicians, intrinsic motivation is found in their profession, but external practice pressures might give rise to burnout. Thus, prevention of burnout and increasing physician wellness become somewhat separate targets that benefit from different, albeit complementary, approaches. This is a priority because physician wellness is a measurable healthcare quality indicator that has well-documented effects at the personal, practice, organizational and societal level (Linzer et al., 2000; Shanafelt, Sloan & Habermann, 2003; Halbesleben & Rathert, 2008; Linzer et al., 2009; Schloss et al., 2009; Wallace & Lemaire, 2007; Wallace, Lemaire & Ghali, 2009; Williams et al., 2010; Salyers et al., 2015) and deserves the same level of attention as institutional financial performance and successful patient care (Wallace, Lemaire & Ghali, 2009; Linzer et al., 2013). Factors that impact physician wellness are influenced by career phase (Linzer et al., 2005; Shanafelt et al., 2009), and, as supported by our findings, physicians in academic medicine contend with issues that are intrinsic to their unique sets of responsibilities. These include the current state of research funding, the restructuring of residency programs, and the need to balance divergent commitments including clinical service, research, teaching, and administration (Shanafelt et al., 2009). At various career stages and in many practice settings, physician leaders and designated mentors can be important facilitators of wellness (Kashiwagi, Varkey & Cook, 2013; Shanafelt et al., 2015). Both physician leaders and mentors were highlighted as important influences on the wellness of participating physicians (Tables 1 and 2). Physician wellness can be aided further by the institution of a physician wellness committee (Spickard, Gabbe & Christensen, 2002; Linzer et al., 2013; Shannon, 2013) such as the one at Stanford (SCPPS), that assesses professional well-being as well as stressors in the workplace, creates programs to prevent and ameliorate burnout, gives physicians a voice, and monitors institutional commitment.

A qualitative study such as this has inherent limitations. One such limitation is the number of participants. These needed to be available at a specific given time, and also be willing to share their thoughts on physician wellness and areas of lack thereof. Even though our number of participants (*n* = 64) was relatively small, the discussed topics were surprisingly similar between individual focus groups, despite the practice environments of diverse medical specialties and differences in departmental culture. Another limitation was that the sessions were not recorded. Although recording is commonly used in focus group studies, recording was carefully considered in advance and feedback on potential recording was obtained. This feedback was overwhelmingly unfavorable, and indicated that participation would be limited—both in attendance and in what was shared—should we proceed with voice recordings. Thus, session recording was not adopted. Two note-takers were present for approximately half of the focus groups sessions, whereas the other half had a single note-taker. This is a deviation from standard methodology and could limit generalizability of the findings. However, comments were read back to participants to ensure accurate reflection of intended meaning, and sessions were ended with a final review of the notes with the group. Bias was further minimized by the fact that physicians had filled out the online survey in advance of the focus group discussion component. This element is a strength of the study that enhances accuracy. Finally, even though this study provides a framework for wellness assessment at practices and institutions in general, such efforts must necessarily be customized to individual settings.

## Conclusions

We probed results from the original Stanford Physician Wellness Survey in greater depth by obtaining physicians’ opinions, in their own words, through focus groups during which physicians’ written responses to five questions were recorded and confidential facilitated discussion based on these questions followed. Investigative aims included physicians’ (a) perceived workplace barriers and facilitators to physician wellness; (b) proposed solutions to work-related wellness barriers; (c) sources of work motivation; and (d) awareness of existing organizational wellness programs. Whereas physicians had limited knowledge of wellness offerings at Stanford, they shared many recommendations for improvement of their wellness in the workplace (Table 2), a proportion of which would require only minor resources. The essence of all suggestions could be summarized in one revealing comment: “I would say, if they can hear anything, then this: help me do my job.” It illustrates that physicians simply wish to be supported in the work they do and hope to achieve facilitation of tasks, in order to deliver outstanding patient care. Such support, together with a meaningful appreciation of their efforts, serves the intrinsic motivating factor of making the best possible contribution to the care of their patients, which is the activity from which, as a group, they derive the greatest professional fulfillment.

## Supplemental Information

10.7717/peerj.1783/supp-1 Supplemental Information 1 Consolidated Criteria for Reporting Qualitative Research—COREQ checklist

## Additional Information and Declarations

### Data Availability

The following information was supplied regarding data availability: The research in this article did not generate any raw data.
