# Supplementary material for: An exploration of key issues and potential solutions that impact physician wellbeing and professional fulfillment at an academic center
Source: PeerJ. 2016 Mar 10;4:e1783. doi: 10.7717/peerj.1783 (PMC4793321; doi:10.7717/peerj.1783)
Supplement: Supplemental Information 1 [file peerj-04-1783-s001.docx]

Domain 1: Research team and reflexivity

Personal Characteristics

1. Interviewer/facilitator. Which author/s conducted the interview or focus group?

Iris Schrijver, MD and Keri Brady, MPH

1. Credentials. What were the researcher's credentials? E.g. PhD, MD

MD and MPH, see above.

1. Occupation. What was their occupation at the time of the study?

Schrijver:

Professor of Pathology and (by courtesy) Pediatrics

Stanford University Medical Center

Brady:

Social Science Research Assistant

Psychiatry & Behavioral Sciences,

Stanford University School of Medicine

1. Gender. Was the researcher male or female?

Both female

1. Experience and training. What experience or training did the researcher have?

MD training, MPH training

Relationship with participants

1. Relationship established. Was a relationship established prior to study commencement?

Iris Schrijver had previously professionally interacted with a few of the participating physicians.

1. Participant knowledge of the interviewer. What did the participants know about the researcher? e.g. personal goals, reasons for doing the research

Reasons for doing the study were explained in the invitation to participate (email) and at the beginning of each focus group.

8. Interviewer characteristics. What characteristics were reported about the interviewer/facilitator? e.g. Bias, assumptions, reasons and interests in the research topic

Reasons for doing the study were explained in the invitation to participate (email) and at the beginning of each focus group.

Domain 2: study design

Theoretical framework

9. Methodological orientation and Theory. What methodological orientation was stated to underpin the study? e.g. grounded theory, discourse analysis, ethnography, phenomenology, content analysis

A multi-method design was applied to conduct a total of 19 focus group sessions in 17 clinical departments. All academic faculty ranks and career lines were represented in the 64 participating physicians, who began the sessions with five open-ended survey questions pertaining to physician wellness in their work environment. Participants entered their answers into a web-based survey program that enabled anonymous data collection. The initial survey component was followed by semi-structured focus group discussion. Data analysis of this qualitative study was informed by the general inductive approach as well as a review of extant literature through September 2015 on physician wellness, professional fulfillment, satisfaction, dissatisfaction, burnout and work-life.

Participant selection

10. Sampling. How were participants selected? e.g. purposive, convenience, consecutive, snowball

A list of all Stanford physicians was utilized as a sampling frame from which initial purposive sampling, stratified by department of all 17 clinical departments, occurred. Additional participants were recruited based on recommendations by physician colleagues approached by the primary investigator (I.S.), a peer physician practicing at Stanford Medicine. Referral to alternative or additional physicians was allowed.

11. Method of approach. How were participants approached? e.g. face-to-face, telephone, mail, email

Invitations to participate in a brief in-person, online survey and a “peer-to-peer focus group” were sent via email to all sampled physicians. Each department was provided with a specific date and time to participate in the study. If they were unable to participate in their department’s designated focus group session, but expressed interest in a separate meeting time, that opportunity was provided.

12. Sample size. How many participants were in the study?

64

13. Non-participation. How many people refused to participate or dropped out? Reasons?

Participating was voluntary; physicians who could not attend frequently did so because of clinical service obligations. If they were unable to participate in their department’s designated focus group session, but expressed interest in a separate meeting time, that opportunity was provided. Three physicians participated by this mechanism and formed a separate focus group, whereas the remaining 61 participants engaged in focus groups arranged per clinical department.

Setting

14. Setting of data collection Where was the data collected? e.g. home, clinic, workplace

Workplace

15. Presence of non-participants. Was anyone else present besides the participants and researchers?

No

16. Description of sample. What are the important characteristics of the sample? e.g. demographic data, date. Data collection

This study was conducted at Stanford University. It included 64 participating physicians from the 17 clinical departments of Stanford Medicine, which includes Stanford Health Care and Stanford Children’s Health, serving the adult and pediatric patient populations, respectively. Physicians from 17 departments (Anesthesia, Dermatology, Medicine, Neurology and Neurological sciences, Neurosurgery, Obstetrics and Gynecology, Ophthalmology, Orthopedic surgery, Otolaryngology, Pathology, Pediatrics, Psychiatry, Radiation Oncology, Radiology, Surgery, Cardiothoracic Surgery, Urology) were eligible for participation if they had an M.D. or D.O degree, if they were in any of the Stanford career trajectories (faculty lines), and if they were members of the medical staff involved in patient care at the adult hospital, the children’s hospital, or both, including associated clinics. Peripheral sites of the greater network of the University Healthcare Alliance (UHA) were excluded. Physicians with trainee status were also excluded.

All ranks and academic faculty lines were represented, with a gender balance of 28 male and 36 female physicians. We recorded only dates and sex.

17. Interview guide. Were questions, prompts, guides provided by the authors? Was it pilot tested?

To encourage independent and unbiased focus group discussions, prior to the start of each forty-five minute focus group discussion, participants were asked to take 15 minutes to independently complete an open-ended, five-question online survey. Anonymous survey responses were collected via Qualtrics (http://www.qualtrics.com). This initial survey component was followed by semi-structured, open-ended discussion about the issues that emerged when participants considered physician wellness at Stanford.

One department was split into two focus groups for concept piloting and location reasons

18. Repeat interviews. Were repeat interviews carried out? If yes, how many?

No

19. Audio/visual recording. Did the research use audio or visual recording to collect the data?

No. In order to stimulate an atmosphere of confidentiality, focus group conversations were not recorded.

20. Field notes. Were field notes made during and/or after the interview or focus group?

In order to stimulate an atmosphere of confidentiality, focus group conversations were not recorded. Instead, qualitative notes were taken by the focus group facilitator (I.S.) and by a research assistant (K. J. S. B.). The jointly documented sessions were followed by immediate debriefing. Confidentiality was maintained by elimination of personally identifying information revealed during the course of the study. The focus group responses were ultimately grouped together so that neither departments, nor individuals could be identified separately.

21. Duration. What was the duration of the interviews or focus group?

One hour

22. Data saturation. Was data saturation discussed?

No

23. Transcripts returned. Were transcripts returned to participants for comment and/or correction?

No, but comments were read back to ensure accuracy.

Domain 3: analysis and findings. Data analysis

24. Number of data coders. How many data coders coded the data?

Anonymous survey responses were collected via Qualtrics (http://www.qualtrics.com). Three researchers (the authors of the manuscript) had access to the data.

25. Description of the coding tree. Did authors provide a description of the coding tree?

No, as it was not applicable. The research flow is depicted in Figure 1.

26. Derivation of themes. Were themes identified in advance or derived from the data?

Derived from the data (see Figure 1)

27. Software. What software, if applicable, was used to manage the data?

Anonymous survey responses were collected via Qualtrics (http://www.qualtrics.com).

28. Participant checking. Did participants provide feedback on the findings?

Not formally, although presentations of the study findings to a subset of the medical staff were made and more are planned.

Reporting

29. Quotations presented. Were participant quotations presented to illustrate the themes / findings? Was each quotation identified? e.g. participant number

Quotations were not identified based on confidentiality, but in Qualtrix they are identified by participant number.

30. Data and findings consistent. Was there consistency between the data presented and the findings?

The data represent our findings.

31. Clarity of major themes. Were major themes clearly presented in the findings?

Yes (Figure 2) and Results section

32. Clarity of minor themes. Is there a description of diverse cases or discussion of minor themes?

Yes (Figure 2) and Results section
